# Once-weekly bortezomib as the standard of care in multiple myeloma: results from an international survey of physicians

**DOI:** 10.1038/s41408-023-00937-0

**Published:** 2023-11-06

**Authors:** Rahul Banerjee, Bo Wang, Larry D. Anderson, Georgia McCaughan, Nikita Mehra, Andrew J. Cowan, S. Vincent Rajkumar, Gurbakhash Kaur

**Affiliations:** 1 Department of Medicine, University of Washington, Seattle, WA USA; 2 Clinical Research Division, Fred Hutchinson Cancer Center, Seattle, WA USA; 3 Willamette Valley Cancer Institute, Eugene, OR USA; 4 Myeloma, Waldenstrom’s, and Amyloidosis Program, Simmons Comprehensive Cancer Center, UT Southwestern Medical Center, Dallas, TX USA; 5 Department of Haematology, St Vincent’s Hospital, Sydney, NSW Australia; 6 Garvan Institute of Medical Research, University of New South Wales, Sydney, NSW Australia; 7 Department of Medical Oncology, Cancer Institute (WIA), Adyar, Chennai, India; 8 Division of Hematology, Mayo Clinic, Rochester, MN USA

**Keywords:** Adverse effects, Myeloma

Dear Editor,

The proteasome inhibitor bortezomib is a key component of treatment regimens for newly diagnosed multiple myeloma (MM). Bortezomib has been dosed twice per week in both industry-sponsored and consortium-run trials of MM induction regimens [1–5]. However, several analyses (Table 1) have demonstrated comparable efficacy with once-weekly versus twice-weekly bortezomib in MM [6–9]. Once-weekly bortezomib has also been associated with lower rates of peripheral neuropathy (PN), with a matched adjusted indirect comparison (MAIC) analysis of randomized controlled trial (RCT) data demonstrating any-grade PN incidences of 32% with once-weekly versus 47% with twice-weekly dosing [7]. MM trial protocols often continue to use twice-weekly bortezomib based under the assumption that dosing schedules from previous trials constitute the standard of care (SOC) for newly diagnosed MM. In contrast, a more accurate definition of SOC regimens involves how typical physicians in the field would approach a given situation [10, 11]. As such, we sought to survey physicians globally to understand their attitudes and perceptions regarding how bortezomib should be dosed.

**Table 1:** Published studies of once-weekly versus twice-weekly bortezomib.

Reference	Overall response rate		Median PFS (95% CI), months	
	Once-weekly	Twice-weekly	Once-weekly	Twice-weekly
Sidana et al. [6]^a^	76.6%	71.4%	NR	NR
Mateos et al. [7]	71.2%	76.1%	19.1 (17.8–21.6)	19.6 (18.8–21.0)
Cook et al. [8]^b^	73.0%	66.0%	36.2 (NR)	38.9 (NR)

Overall response rates and median progression-free survival are depicted for analysis. When multiple regimens or dosing routes were included, unadjusted comparisons with subcutaneous dosing of once-weekly versus twice-weekly bortezomib (assuming 21-day cycles) are shown.

*CI* confidence interval, *NR* not reported, *PFS* progression-free survival.

^a^Included patients with AL amyloidosis.

^b^Very good partial response or better.

We conducted a global online survey of hematologists/oncologists who had treated at least 1 patient with MM in the past 12 months. The anonymous survey, which was available in English from June to September 2023, was distributed via social media, targeted emails, and professional society outreach. The 14-question survey (Supplemental Table 1) included self-reported demographic questions as well as questions about awareness, usage, benefits, disadvantages (including numeric estimates of PN incidences), and specific barriers regarding bortezomib. Given that the field has consistently moved from intravenous to subcutaneous bortezomib given lower rates of PN with the latter [12], we also asked about barriers to the adoption of subcutaneous bortezomib to provide exploratory comparisons. An optional field was provided for anonymous comments. Results were compared descriptively using medians and interquartile ranges (IQR). Where applicable, Fisher’s exact tests or Wilcoxon tests were used. This study was declared exempt by the University of Washington Institutional Review Board.

Of 340 webpage visits, 217 responses were recorded corresponding to a 64% completion rate. Over a third of respondents (38%, *n* = 83) practiced in community settings, and 59% (*n* = 127) had treated >20 patients with MM in the past year. With regard to years of clinical experience, responses were 1–4 years in 35% (*n* = 75), 5–10 years in 30% (*n* = 66), and >10 years in 35% (*n* = 76). Most respondents were from United States (US) academic practices (25%, *n* = 55), US community practices (24%, *n* = 51), Australian academic practices (6%, *n* = 13), or Indian academic practices (5%, *n* = 11). However, responses were recorded from 38 countries across 6 continents (Supplemental Table 2). Respondents from low- or middle-income countries (LMIC), as defined by the World Bank [13], comprised 29% of responses (*n* = 62). Forty-five percent of respondents (*n* = 98) had previously helped write institutional or societal guidelines, including 21 International Myeloma Working Group members.

Respondents reported using once-weekly bortezomib for a median of 95% of their own patients (IQR 80–100%) and almost always used subcutaneous bortezomib (median 100% of patients, IQR 100–100%). Minorities of respondents reported using twice-weekly more frequently than once-weekly bortezomib (12%, *n* = 25) or intravenous more frequently than subcutaneous bortezomib (5%, *n* = 10). There were no significant differences based on practice type (95% academic versus 95% community-based, *p* = 0.81), patient volume (95% if >20 MM patients versus 95% if ≤20 MM patients, *p* = 0.60), or guideline-writing experience (95% if any versus 92% if none, *p* = 0.20). There were no significant differences based on countries where respondents practiced: 95% for US-based physicians versus 94% for non-US-based physicians (*p* = 0.22) and 92% for LMIC-based physicians versus 95% for non-LMIC-based physicians (*p* = 0.68). Within the US, responses were similar for academic versus community-based physicians (95% versus 95%, *p* = 0.19).

As shown in Table 2, large majorities felt that once-weekly bortezomib leads to comparable durations of response (80%), is associated with less PN (90%), and is preferred by patients (93%) compared to twice-weekly bortezomib. The only scenario where once-weekly bortezomib was not broadly preferred was acute cast nephropathy, where 62% of respondents felt that twice-weekly bortezomib was superior. In terms of their understanding of the scientific literature, 59% (*n* = 127) were aware of ≥ 1 study showing comparable progression-free survival with once-weekly versus twice-weekly bortezomib; 12% (*n* = 26) were not aware of any such studies and the remainder were unsure. Similarly, 63% (*n* = 136) were aware of studies showing less PN with once-weekly bortezomib while 5% (*n* = 11) were not aware and the remainder were unsure. Respondents estimated the incidence of any-grade PN to be significantly lower (*p* < 0.001 by signed-rank testing) with once-weekly bortezomib (median 30%, IQR 20–43%) than twice-weekly bortezomib (median 50%, IQR 40–73%).

**Table 2:** Physician attitudes regarding once-weekly bortezomib.

Compared to twice-weekly bortezomib, once-weekly bortezomib….	Agree		Disagree		Unsure	
	%	(*n*)	%	(*n*)	%	(*n*)
Has comparable durations of responses.	80%	(171)	5%	(11)	15%	(33)
Has lower rates of peripheral neuropathy.	90%	(194)	4%	(8)	6%	(14)
Is generally preferred by patients.	93%	(201)	2%	(5)	4%	(9)
Has inferior pharmacokinetics.	18%	(39)	45%	(97)	37%	(80)
Is harder to get regulatory approval for.	7%	(16)	55%	(118)	38%	(81)
Is inferior in acute cast nephropathy.	62%	(133)	16%	(34)	22%	(48)

Missing responses for any given question are not included.

Reported barriers to ordering once-weekly or subcutaneous bortezomib are summarized in Supplemental Table 3. The strongest reported barrier was a perceived lack of prospective data (30%, *n* = 65), while difficulty modifying treatment orders was reported as a barrier by 23% (*n* = 49). Finally, 13% (*n* = 28) cited resistance from pharmacist colleagues who preferred specific trial-studied regimens. In comparison, 11% (*n* = 23) of respondents felt that subcutaneous bortezomib lacked prospective data while 6% (*n* = 14) cited difficulties with modifying treatment orders. Representative comments (*n* = 46 comments altogether) are summarized in Supplemental Table 4. Five respondents highlighted the time-limited role of twice-weekly bortezomib for acute cast nephropathy. Six respondents specifically expressed frustration with continued twice-weekly dosing in clinical trials, with one respondent stating, “The FDA [Food and Drug Administration] needs to allow trials to utilize once-weekly [bortezomib] so that the clinical trial data matches real-world practice.”

In summary, we surveyed 217 physicians from 38 countries regarding how and why they order bortezomib in MM. We found that once-weekly subcutaneous bortezomib was overwhelmingly preferred regardless of practice setting or country, with at least 90% of respondents preferring once-weekly dosing in each subgroup of interest. Consistent with several previous studies [6–9], large majorities of respondents felt that once-weekly bortezomib was associated with comparable responses and less neuropathy. Specific estimated PN incidences were quite similar to the MAIC rates of 46.7% (twice-weekly) versus 32.1% (once-weekly) compiled from a previously published analysis of prospective RCT-derived data [7]. Given the need for rapid light chain reduction in acute cast nephropathy [14], this was the only scenario where respondents preferred twice-weekly bortezomib. Interestingly, although neither bortezomib dosing frequency nor bortezomib route of administration have been studied head-to-head in RCTs, respondents were only a third as likely to cite a lack of prospective data as a barrier to implementing subcutaneous bortezomib dosing.

Study limitations include selection bias given our reliance on electronic methods to disseminate an English-language survey. We also did not investigate physician perceptions regarding cycle lengths. However, in one previous study, previous research suggests that once-weekly bortezomib performs similarly whether dosed in 21-day or 28-day cycles [8]. Strengths of our study include the breadth of responses spanning 6 continents, including 83 community-based oncologists and 62 physicians who practiced in LMICs. Most respondents were aware of studies demonstrating comparable efficacy and reduced toxicities with once-weekly bortezomib, and specific estimates of PN were relatively accurate compared to published data. Given that how physicians would typically manage their own patients is a key pillar of defining SOC paradigms [10, 11], we conclude that most physicians across the globe prefer once-weekly bortezomib as a standard-of-care regimen for MM induction. Based on the strong support for once-weekly bortezomib in our study, an RCT of once-weekly bortezomib versus twice-weekly bortezomib would be impractical to conduct given the evident lack of equipoise to justify the use of twice-weekly bortezomib.

The next steps for our group involve standardizing this SOC both in practice and in trials. The most commonly reported barrier to using once-weekly bortezomib in our study was a lack of prospective data, which is not strictly accurate given published secondary analyses from randomized clinical trials [7]. With regard to modifying treatment orders, we recommend referencing the 28-day “RVD premium lite” regimen with once-weekly bortezomib [15]. Thirteen percent of respondents reported resistance from colleagues who prefer a trial-studied regimen with once-weekly bortezomib. Several physicians expressed their wish that the FDA would “allow” trials of once-weekly bortezomib, a belief driven by perceived fears that regulatory agencies would reject arms containing once-weekly bortezomib as non-SOC regimens. In fact, both the ALCYONE and BOSTON trials (leading to the FDA approvals of daratumumab/bortezomib/melphalan/prednisone and of selinexor/bortezomib/dexamethasone, respectively) did incorporate once-weekly bortezomib into their experimental arms [16, 17]. Furthermore, FDA guidance around informed consent has stipulated that the SOC is “evidenced by publication in a peer-reviewed journal or recognition by a professional medical society.” [18] Given the overwhelming consensus favoring once-weekly bortezomib in this peer-reviewed publication, we encourage future clinical trials to use the results of this study to justify once-weekly bortezomib as a SOC strategy to reduce PN and to improve the patient experience during treatment for MM.

### Supplementary information

Supplemental materials

## Data Availability

Data are available from the corresponding author upon reasonable request.
