# Supplementary material for: Once-weekly bortezomib as the standard of care in multiple myeloma: results from an international survey of physicians
Source: Blood Cancer J. 2023 Nov 6;13(1):162. doi: 10.1038/s41408-023-00937-0 (PMC10625982; doi:10.1038/s41408-023-00937-0)
Supplement: Supplementary file 1 — Supplemental materials [file 41408_2023_937_MOESM1_ESM.docx]

**Supplemental Table 1: Survey questions**

| **Question** | **Response type** |
| --- | --- |
| 1. How many patients with multiple myeloma (MM) have been under your care in the past 12 months? | Dropdown list. |
| 2. When you order bortezomib (Velcade®), approximately how often do you order ONCE-weekly bortezomib as opposed to twice-weekly bortezomib? | Percentage slider from 0-100% |
| 3. When you order bortezomib (Velcade®), approximately how often do you order subcutaneous (subQ) bortezomib as opposed to intravenous (IV) bortezomib? | Percentage slider from 0-100% |
| 4. Compared to twice-weekly bortezomib, regimens with ONCE-weekly bortezomib...   - have similar durations of responses - have less any-grade peripheral neuropathy - are preferred by patients | Multiple choice (disagree, agree, unsure / neutral) for each question |
| 5. Compared to once-weekly bortezomib, regimens with TWICE-weekly bortezomib are...   - Clinically superior due to better pharmacokinetics - More likely to get insurance authorization - More appropriate in acute cast nephropathy | Multiple choice (disagree, agree, unsure / neutral) for each question |
| 6. Do any of these factors pose barriers to ordering ONCE-weekly bortezomib for your own patients?   - Lack of prospective evidence for efficacy - Difficulty modifying treatment order sets - Resistance from pharmacy or nursing colleagues - Lost clinic revenue from fewer visits | Multiple choice (not a barrier, mild barrier, strong barrier, unsure) for each question |
| 7. What are the approximate rates of peripheral neuropathy (PN) in each of these scenarios with subQ bortezomib?   - % any-grade PN with ONCE-weekly bortezomib - % any-grade PN with TWICE-weekly bortezomib - % Grade 3+ PN with ONCE-weekly bortezomib - % Grade 3+ PN with TWICE-weekly bortezomib | Percentage slider from 0-100% for each question |
| 8. How many studies of bortezomib, including non-randomized trials and retrospective analyses, are you aware of that have shown the following?   - Once-weekly bortezomib has less neuropathy than twice-weekly - Once-weekly and twice-weekly bortezomib have similar efficacy - SubQ bortezomib has less neuropathy than IV bortezomib - SubQ and IV bortezomib have similar efficacy | Multiple choice (No studies, 1 study, 2+ studies, unsure) for each question |
| 9. Do any of these factors pose barriers to ordering subQ bortezomib (as opposed to IV)?   - Lack of prospective evidence - Not available in my country - Too expensive or can’t get approved - Difficulty modifying treatment order sets | Multiple choice (not a barrier, mild barrier, strong barrier, unsure) for each question |
| 10. Have you ever served on any guideline committees focused on multiple myeloma?   - Yes: as part of IMWG recommendations - Yes: as part of a non-IMWG professional society - Yes: at a regional or national level - Yes: at a local or institutional level - No: I have not served on a guideline committee | Yes/no checkboxes for each question. |
| 11. In what country do you primarily practice? | Dropdown list. |
| 12. In what setting do you primarily practice? | Dropdown list. |
| 13. How many years have you been practicing since the completion of your training? | Dropdown list. |
| 14. Any optional comments that you'd like to share? Please do not provide any identifying information. | Free text |

**Supplemental Table 2: Countries in which Physicians Practiced**

| **North America** |  |  | **Africa** |  |  |
| --- | --- | --- | --- | --- | --- |
| Canada | 3 | (1.4%) | Egypt* | 1 | (0.5%) |
| Mexico* | 2 | (0.9%) | Morocco* | 1 | (0.5%) |
| United States | 106 | (48.9%) | Tunisia* | 1 | (0.5%) |
| **South America** |  |  | **Asia** |  |  |
| Argentina* | 1 | (0.5%) | China* | 1 | (0.5%) |
| Brazil* | 3 | (1.4%) | India* | 14 | (6.5%) |
| Chile | 3 | (1.4%) | Israel | 1 | (0.5%) |
| Colombia* | 2 | (0.9%) | Japan | 1 | (0.5%) |
| Ecuador* | 3 | (1.4%) | Lebanon* | 1 | (0.5%) |
| Paraguay* | 3 | (1.4%) | Malaysia* | 1 | (0.5%) |
| Peru* | 1 | (0.5%) | Pakistan* | 10 | (4.6%) |
| Uruguay* | 1 | (0.5%) | Saudi Arabia | 2 | (0.9%) |
| Venezuela* | 2 | (0.9%) | Sri Lanka* | 1 | (0.5%) |
| **Europe** |  |  | Syria* | 1 | (0.5%) |
| Belgium | 1 | (0.5%) | Thailand* | 1 | (0.5%) |
| France | 2 | (0.9%) | Turkey* | 10 | (4.6%) |
| Germany | 2 | (0.9%) | **Australia** |  |  |
| Greece | 1 | (0.5%) | Australia | 17 | (7.8%) |
| Norway | 1 | (0.5%) | New Zealand | 1 | (0.5%) |
| Poland | 1 | (0.5%) |  |  |  |
| Spain | 2 | (0.9%) |  |  |  |
| Ukraine* | 1 | (0.5%) |  |  |  |
| United Kingdom | 10 | (4.6%) |  |  |  |

Starred countries are defined as low- or middle-income countries by the World Bank.^[[1]](#footnote-2)^

**Supplemental Table 3: Reported barriers to once-weekly or subcutaneous bortezomib**

| ***Do these factors pose barriers to ordering*** | | **Not a barrier** | | **Barrier*** | | **Unsure** | |
| --- | --- | --- | --- | --- | --- | --- | --- |
| ***once-weekly or subcutaneous bortezomib?*** | | % | (n) | % | (n) | % | (n) |
| *(Once-weekly)* | Lack of prospective data | 67% | (144) | 30% | (65) | 3% | (7) |
| *(Once-weekly)* | Difficulty modifying orders | 77% | (165) | 23% | (49) | 0% | (0) |
| *(Once-weekly)* | Pharmacists prefer trial regimen | 87% | (188) | 13% | (28) | 0% | (1) |
| *(Once-weekly)* | Lost clinic revenue | 93% | (201) | 5% | (10) | 2% | (5) |
| *(Subcutaneous)* | Lack of prospective data | 87% | (188) | 11% | (23) | 3% | (6) |
| *(Subcutaneous)* | Difficulty modifying orders | 92% | (198) | 6% | (14) | 2% | (4) |
| *(Subcutaneous)* | Too expensive | 94% | (201) | 5% | (10) | 1% | (3) |
| *(Subcutaneous)* | Not available in country | 95% | (206) | 3% | (7) | 2% | (4) |

Missing responses for any given question are not included.

* Groups respondents who said selected “mild barriers” and “strong barrier.”

**Supplemental Table 4: Representative Comments by Physicians**

| **In favor of using once-weekly bortezomib** | ***n*** |
| --- | --- |
| Importance of avoiding even Grade 1 PN, which can affect patient QOL | 2 |
| Enhanced convenience to patients and/or less “time toxicity” | 4 |
| Better tolerated for patients with concurrent AL amyloidosis | 1 |
| **Barriers to using once-weekly bortezomib** | ***n*** |
| Acute cast nephropathy during first cycle | 5 |
| Belief that twice-weekly dosing deepens responses before ASCT | 1 |
| **In favor of using subcutaneous bortezomib** | ***n*** |
| Importance of avoiding even Grade 1 PN, which can affect patient QOL | 2 |
| Enhanced convenience to patients, e.g. via “chemo bus” administration | 1 |
| **Barriers to using subcutaneous bortezomib** | ***n*** |
| In several countries, bortezomib vials labeled “for IV use only” | 3 |
| Rare patients with recurrent injection site reactions | 1 |
| **Other relevant comments** | ***n*** |
| Frustration with clinical trials largely requiring twice-weekly bortezomib | 6 |
| Other factors (specific bortezomib dose, comorbidities) also affect PN risk | 5 |
| Brand-name bortezomib much more expensive than generic bortezomib | 4 |
| Difficulties with bortezomib wastage given fixed vial sizes | 1 |

Some comments are listed in more than one category depending on their contents.

Abbreviations: ASCT, autologous stem cell transplantation; IV, intravenous; PN, peripheral neuropathy; QOL, quality of life.

1. World Bank. World Bank Country and Lending Groups. <https://datahelpdesk.worldbank.org/knowledgebase/articles/906519>. Accessed 2023 Sep 21. [↑](#footnote-ref-2)
